# A Smart and Balanced Energy-Efficient Multihop Clustering Algorithm (Smart-BEEM) for MIMO IoT Systems in Future Networks †

**DOI:** 10.3390/s17071574

**Published:** 2017-07-05

**Authors:** Lina Xu, Gregory M. P. O’Hare, Rem Collier

**Affiliations:** 1 School of Computer Science, University College Dublin, Belfield, Dublin 4, Ireland; gregory.ohare@ucd.ie (G.M.P.O.); rem.collier@ucd.ie (R.C.); 2 CONSUS (Crop OptimisatioN through Sensing, Understanding & viSualisation), University College Dublin, Belfield, Dublin 4, Ireland

**Keywords:** WSN, MIMO, Energy Efficiency, QoE, Clustering, IoT, Future Network, 5G

## Abstract

Wireless Sensor Networks (WSNs) are typically composed of thousands of sensors powered by limited energy resources. Clustering techniques were introduced to prolong network longevity offering the promise of green computing. However, most existing work fails to consider the network coverage when evaluating the lifetime of a network. We believe that balancing the energy consumption in per unit area rather than on each single sensor can provide better-balanced power usage throughout the network. Our former work—Balanced Energy-Efficiency (BEE) and its Multihop version BEEM can not only extend the network longevity, but also maintain the network coverage. Following WSNs, Internet of Things (IoT) technology has been proposed with higher degree of diversities in terms of communication abilities and user scenarios, supporting a large range of real world applications. The IoT devices are embedded with multiple communication interfaces, normally referred as Multiple-In and Multiple-Out (MIMO) in 5G networks. The applications running on those devices can generate various types of data. Every interface has its own characteristics, which may be preferred and beneficial in some specific user scenarios. With MIMO becoming more available on the IoT devices, an advanced clustering solution for highly dynamic IoT systems is missing and also pressingly demanded in order to cater for differing user applications. In this paper, we present a smart clustering algorithm (Smart-BEEM) based on our former work BEE(M) to accomplish energy efficient and Quality of user Experience (QoE) supported communication in cluster based IoT networks. It is a user behaviour and context aware approach, aiming to facilitate IoT devices to choose beneficial communication interfaces and cluster headers for data transmission. Experimental results have proved that Smart-BEEM can further improve the performance of BEE and BEEM for coverage sensitive longevity.

## 1. Introduction

In this section, we provide the background study for our work. Firstly, we illustrate clustering techniques for Wireless Sensor Networks (WSNs). Then we discuss the trend of transformation from WSNs to Internet of Things (IoT). Thirdly, we clarify the need for clustering, and corresponding challenges of applying such a technique for IoT systems, in Multiple-In-Multiple-out (MIMO) featured 5G environments. Finally, a brief idea of our proposed solution and the structure of this paper are presented.

### 1.1. Clustering for WSNs

**Figure 1:**
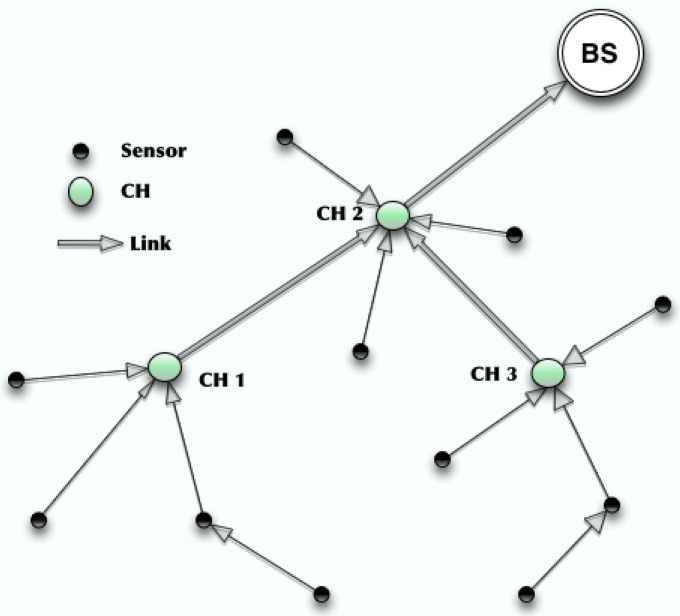
An Example of Cluster-Based WSN.

Wireless Sensor Networks (WSNs) are networks composed of distributed micro-devices embedded with various sensing abilities (called sensors), which are used to monitor the environment and send the information back to a central Base Station (BS) [1,2]. WSNs typically include a large number of sensors that are equipped with limited energy resources, but are required to operate without recharging or replacing batteries for extended periods of time. Energy efficient communication solutions for WSNs are highlighted by many researchers [3,4]. In order to prolong a networks longevity, clustering techniques have been introduced to achieve energy efficient communication between sensors. A clustering algorithm can partition sensors into different clusters/groups, as shown in Figure 1. In each cluster, a Cluster Header (CH) is elected to be in charge of generating a transmission schedule, gathering data from all the sensors in the cluster and transmitting the assembled data back to the BS. Based on the clustered structure, the system can maintain a longer life by scheduling the duty cycle between the sensors within a cluster, without harming the functionality of the network. In addition to saving energy from scheduling, a sensor can also reduce energy consumption from communication since it only needs to communicate with a local CH rather than a far located BS. Clustering techniques can decompose sensors within a WSN into different clusters. After joining a cluster, a sensor normally only needs to communicate with its own CH. A CH can communicate with the BS directly or through other CHs, as shown in Figure 1. The routing between the sensors in the same cluster is called *intra-cluster routing*. The routing between the CHs and the BS is called *inter-cluster routing*. The routing scheme can be either single hop or multihop, and is dependent upon several factors, such as the objectives of a clustering algorithm or the communication capability of the sensors.

### 1.2. From WSNs to IoT

WSNs were originally proposed for military surveillance purposes [5]. Due to its early success, this technology was then envisioned for use in applications such as habitat monitoring [6], weather monitoring [7], agriculture monitoring [8] and wildlife monitoring [9]. The revolution that is necessary to ensure WSN technologies flourish in a similar manner requires more effort in simplifying design, implementation, deployment and usability. The societal impact of WSNs is highly influenced by the number of the users, which is itself determined by the quality and the quantity of the available applications. Currently, there is an urgent need to inspire developers to build more useful Over The Top (OTT) applications to improve people’s life quality or experience. Therefore, the concepts of Internet of Things (IoT) [10] and Internet of Everything (IoE) [11] have been proposed and popularised through concepts such as smart buildings/home, smart cities, smart roads and connected cars [12,13]. WSNs came first, providing the enabling technological foundations for IoT.

The fundamental difference between WSNs and IoT is that the dynamics and diversities in IoT are much higher than that in WSNs, which include the following aspects:

1. **Applications:** Traditional WSN applications are mainly focusing on monitoring the environment and then collecting the data. The sensors are deployed at fixed locations in the field. The volume of the data transmitted in the network is small. However, IoT applications can range from smart kitchen to smart city monitoring. All the applications in these systems have specific requirements on the network performance. For instance, the smart road lighting application in a smart road system requires short delays. The communication for the cameras in smart monitoring systems requires high bandwidth.
2. **IoT devices:** WSN systems normally have homogeneous sensors, in terms of sensing ability, processing capability and power supply. This feature has simplified the design of the relevant protocols, such as routing protocols. However, in IoT systems, this feature is no longer applied. For example, in a smart home system, the fridge, the vacuuming robot and the light sensing devices all have their own functionalities and characteristics in this system. Furthermore, the devices in IoT systems are no longer limited to those basic sensors.
3. **Communication ability:** Since the features of the connected devices in IoT systems vary a lot, their communication capabilities including communication range, communication band and communication power consumption are all different.
4. **Number of connected devices:** By the end of 2020, according to Cisco, there will be over 50 billion connected devices [11]. Managing such a large number of devices along with the generated data is challenging in IoT systems.
5. **Objectives:** As mentioned, WSN systems are mainly used for monitoring the environment and collecting data. User profile and system context are rarely discussed. One of the most important objectives for IoT systems is to improve people’s life and their personal experience. User oriented and context aware design is demanded in IoT systems.

From the communication perspective, each IoT device may be embedded with multiple radio access interfaces enabling Multiple-In and Multiple-Out (MIMO) communication. Network selection among the available interfaces has drawn many attentions from both the 3GPP organisations and researchers [14]. However, traditional clustering strategies for WSNs typically assume the sensors in the networks are homogeneous with only a single communication interface available. If a heterogeneous network is considered, the degree of the diversity is still rather low (For example, some heterogeneous networks simply assume that some sensors have doubled power supplies than others). This assumption has become unrealistic recently, especially in IoT systems. The challenges when migrating IoT systems to 5G platforms have been discussed a lot from a general perspective [15]. In the next section, we will show the necessity of clustering techniques in IoT systems that are based on 5G networks.

### 1.3. Clustering for IoT in 5G Scenarios

Currently, the 3GPP organisations, mobile operators and academics are trying to realise 5G by the end of 2020. 5G, which is an enabler for Machine-to-Machine (M2M) type communication, has features such as 1 to 10 Gbps speed, 1 millisecond latency, 100% coverage and reliability. It aims to provide support for good Quality of user Experience (QoE) for a large range of applications and usages. Cellular involved backhaul is becoming a popular approach adopted by many systems in order to provide full connectivity. A novel two-stage re-clustering algorithm has been introduced in [16] to reduce high load on cells in hotspot areas and in turn to improve user satisfaction. Smart coordinating multiple access points can improve the overall spectral efficiency.

In such a 3GPP involved IoT backhaul, we indicate that clustering techniques are still necessary and beneficial for the following reasons:

1. **Energy efficiency:** In IoT systems, many sensors/devices are still deployed requiring a lifetime measuring in terms of years. 5G aims to provide M2M communication, allowing devices to have up to 10 years’ battery life. Therefore, energy efficiency still is a challenging problem in IoT systems regarding of Quality of Service (QoS) [17]. In addition, from the perspective of green computing, with 50 billion connected devices, if every single device could reduce 1% energy consumption, it can significantly reduce the total electricity bill.
2. **Distributed processing:** In the last 10 years, big data has been a really hot topic and people generally expect treasures in the data. However, not all the data is useful. Treasure hunting in massive amounts of meaningless data can be costly. Furthermore, data transmission through the network and data maintaining on the server are also expensive. It is essential to filter out worthless or redundant data from the source, rather than transmitting it back to the core network. This task can be assigned to CHs.
3. **Management hierarchy:** As we have discussed, the major difference between WSNs and IoT is the diversities—from the devices themselves to the OTT applications. Besides, with such a large number of devices, an extensible and dynamic hierarchical structure can achieve effective and efficient management.

### 1.4. Our Proposed Solution—BEE(M) and Smart-BEEM

In this paper, we propose a Balanced Energy-Efficient clustering algorithm, named as BEE. It can elect CHs according to both energy consumption and sensor distributions. It not only extends the networks longevity, but also maintains the network coverage. We also provide another version of BEE, called Multihop BEE (BEEM), which can support multihop inter-cluster communication. BEEM can further improve the network coverage comparing with BEE through reducing high energy consumption and long distance inter-cluster communication. Smart-BEEM is an extended work based on BEEM. Firstly, it can elect CHs to extend the coverage sensitive longevity. Secondly, it can select communication interface based on the user scenarios, including the available interfaces, CHs in range and user applications. By considering those factors, Smart-BEEM can further extend the coverage sensitive longevity without scarifying the user utility.

The rest of this paper is organised as follows: The related work is introduced in Section 2. The motivation for BEE(M) and Smart-BEEM is delivered in Section 3. The detailed design for BEE(M) is provided in Section 4. Based on BEEM, algorithm Smart-BEEM is proposed and analysed in Section 5. The construction of the experimental environment for evaluation is given in Section 6 and the experimental results are illustrated in Section 7. Future work is discussed in Section 8. Finally the conclusions of this paper are drawn in Section 9.

## 2. Related Work

Low-Energy Adaptive Clustering Hierarchy (LEACH) [18] provides an elegant clustering routing approach that has inspired many varied solutions. After the CHs are randomly selected, they will broadcast their information to all the sensors in the network. Based on the received information, each sensor decides which CH it wants to join. Instead of using random CH election scheme, Hybrid Energy-Efficient Distributed (HEED) [19] can select the sensors with high battery levels to be CHs through the proposed iteration CH election scheme. In the iteration CH election scheme, CHs are classified into two types: tentative and final CHs. Some initial CHs are selected randomly as tentative CHs. After each iteration, every sensor increases their probabilities to become a CH. In this algorithm, they set this probability to be the CH self election threshold and the threshold value is 1. Since the probability is increased twice after each iteration, once the probability is 1, the sensor will claim itself as a final CH. For example, initially this probability for a sensor to become a CH is 0.3. After the first iteration, it increases to 0.6, and then after the second iteration it increases to 1 (the real value is 1.2 but cut to 1). A tentative CH will give up being a CH if it discovers a final CH in its communication range. The sensors are not covered by any final CHs will elect themselves to be CHs. This iteration CH election scheme guarantees that the probability of the phenomena that two sensors, within each other’s communication range, both become CHs is rare. Therefore, it is can be deduced that the CHs are well distributed over the network and thus all the clusters have similar size. HEED involves significant overhead due to the heavy broadcast in each iteration. As the number of iterations required in the initial phase increases, the overhead and network delays are also increased. LEACH and HEED are two typical Voronoi structure based clustering algorithms that have inspired many other algorithms. DWEHC [20] made an improvement on HEED by using sensors’ location information. It can achieve more balanced cluster size, and consequently achieve more balanced power consumption on each sensor. However, the location information of the sensors used in this algorithm is not always available. DWEHC also improved HEED by supporting multihop inter-cluster and multihop intra-cluster communication.

In PEGASIS [21], every sensor in the network transmits its data to one of its neighbours. In this way, the gathered data is transferred from one node to another through a chain. A designated node (a node receives data from both sides) will send the assembled data back to the BS. PEGASIS is claimed as a distributed algorithm. However, every single node needs to obtain the global topology map of the network.

CCS [22] is a centralised clustering algorithm based on PEGASIS. Instead of using a single chain structure, CCS utilises a multihop chain structure. Regarding the BS as the centre of the network, each sensor assigns itself a level number according to the signal strength received from the BS. Through this way, the sensors in the network are organised into a hierarchical structure. For each level, the sensors perform transmission and fusion in the same way as that in PAGASIS. The sensor that is elected as the CH will gather data from all the other sensors on the same level and then transmit the assembled data to the CH in the 1-lower level. Once being assigned a level, a sensor will not change its level unless the location of the BS changes. This structure suffers from a problem that the sensors near the BS can die soon from forwarding packets for the sensors in the higher levels. Only total power consumption of the network is measured to evaluate this algorithm. There is no evidence showing a balanced power usage throughout the network.

In a spectrum structure based network, the sensors are partitioned based on both the distance and the angle to the BS. The angle is captured from a scanning sweep from the BS at a specific time. S-WEB [23] is a spectrum structure based clustering algorithm. The first step in S-WEB is similar to CCS. All sensors are partitioned into layers based on their distance (measured but signal strength) to the BS. Then the BS does a 360-degree scanning sweep by sending out signal at one angel at a specific time. Sensors are clustered into cells based on the layer number and scanning angle. In each cluster cell, the sensor with the highest residual energy will be elected as the CH. All the sensors are responsible for forwarding packets. The cluster structure of the network is fixed after performing S-WEB. It is not adaptive to the dynamic changes in the network, like node failure. The evaluation of S-WEB is only based on the comparison with a non-cluster routing solution—Direct Routing.

In Table 1, we summarise the characteristics of 6 existing clustering algorithms. Few of the clustering algorithms consider network coverage.

Furthermore, we have reviewed most of the existing survey papers for clustering in WSNs for the last decade. There are many overlapping studies and investigations. Many of them lack deep analysis and comprehensive introduction. These four selected survey papers shown in Table 2 ([24,25,26,27]) are good to start with when researchers are about to explore in this area. The selected survey papers in Table 2 include a large number of clustering algorithms. There are also some new studies in recently two years (2015 and 2016) on clustering that are not covered by the existing reviewing papers, such as RINtraR [28], SenCar [29], FL-LEACH [30], BEEM [31] and PathQuality [32]. The common analysis topics include: convergence time, node mobility, cluster overlapping, location awareness, energy efficiency, failure recovery, balanced cluster/cluster size, cluster stability, cluster count, load balancing, deliver delay, intra/inter routing schemes, objectives and complexity, etc. However, besides energy efficiency, other discussed metrics are mostly about network topology.

As we can see, most existing studies are for transitional WSNs and limited work has shown interests in highly diverse IoT systems. Even fewer clustering algorithms consider the impact from 5G communication, especially the MIMO communication features. Communication abilities of different sensors or IoT devices vary greatly in IoT networks. The heterogeneous networks we are talking nowadays are no longer limited to the scenarios with just several super nodes. Supporting context aware clustering is missing in such a context.

## 3. Motivation

Based on the analysis on existing work for clustering in Section 2, we can see that two challenges need to be addressed. One is how to extend networks lifetime and meanwhile maintain networks coverage. The other one is how to adapt to and utilise the MIMO feature enabled in 5G scenarios. This section clarifies our motivation from two perspectives: (1) balanced energy consumption and (2) context awareness in 5G networks.

### 3.1. Motivation for Balanced Energy Efficiency

**Figure 2:**
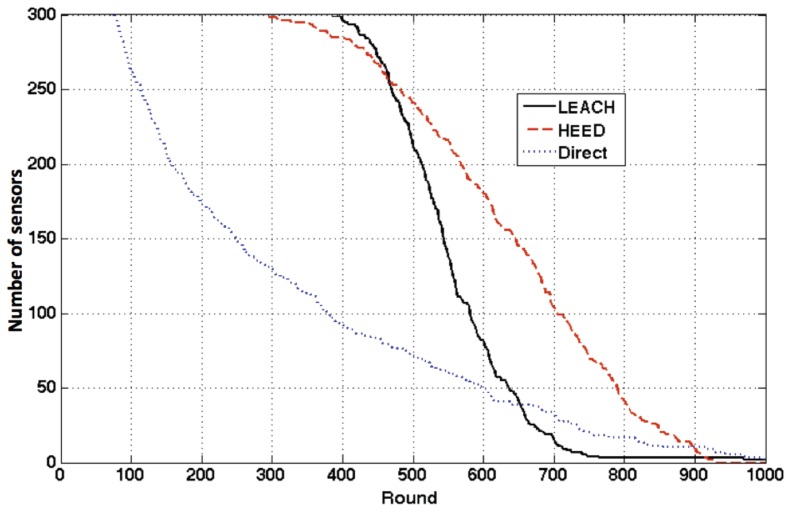
Number of sensors still alive in the network.

Most existing clustering algorithms focus on maintaining the number of sensors that are still alive to extend the longevity, ignoring the distribution of the sensors. The coverage of a network is highly determined by the sensor distribution and it is crucial in most systems, like wildlife monitoring or battlefield sensing. Most existing work assumes that a sensor’s sensing range is the same as its communication range. Therefore, the sensing coverage area is the same as communication area. Once the network is still connected, the network coverage is also guaranteed. This can simplify the connectivity problem and the coverage problem into one. However, this assumption is not true in reality. A sensor or an IoT device can have short distance communication and also long communication abilities. Even for classic clustering algorithms like LEACH and HEED, they only discussed communication connectivity problem, ignoring the fact that the network coverage could change during the network operations. To expose the network coverage problem in current work, three communication schemes are examined: direct routing, general LEACH and HEED. The experiment settings are the same as in HEED, shown in Table 9 in Section 6 Experimental Construction. In HEED, each CH is set to use a single hop to communicate with the BS. The number of nodes still alive after each round in the network is shown in Figure 2. LEACH and HEED performed significantly better than the direct transmission approach. However, the comparison between LEACH and HEED requires careful analysis. The first node died earlier in HEED than that in LEACH. After the first node died in LEACH, all the sensors run out of power in a short period of time. Therefore, the last node died later in HEED than that in LEACH. Depends on the definition for network lifetime (from the system starts till the first or the last node dies), LEACH and HEED can have different performances.

**Figure 3:**
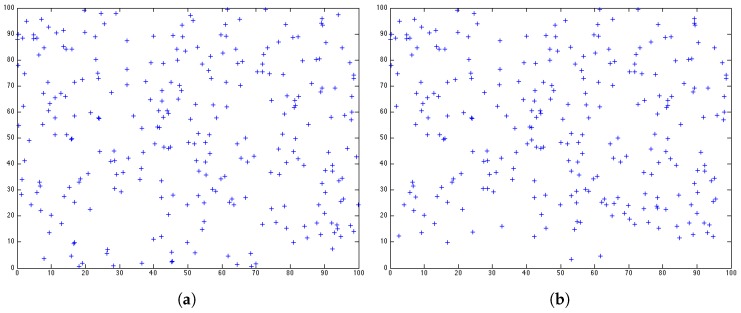
Distribution of nodes still alive after 450 rounds. (**a**) LEACH; (**b**) HEED.

However, in this paper, we aim to evaluate the network lifetime from another perspective. We argue that the number of nodes left in the network cannot present the QoS of a system. In Figure 2, around 450 round, we can see that LEACH and HEED almost have the same number of nodes in the network. The snapshots of the sensor distribution at 450 round for both protocols are shown in Figure 3. With the same number of sensors left in the network, we can see that LEACH has a better distribution as the sensors still alive can cover a slightly larger area than HEED. Depending on the emphasis of a system, the way to measure the lifetime of a network is not unique. When the first node or the last node dies is not the most important issue in term of the network coverage. The coverage of the network should be considered when measuring the lifetime from a QoS perspective. Since different systems have different requirements, simply evaluating the network lifetime by the number of sensors is not convincing.

**Figure 4:**
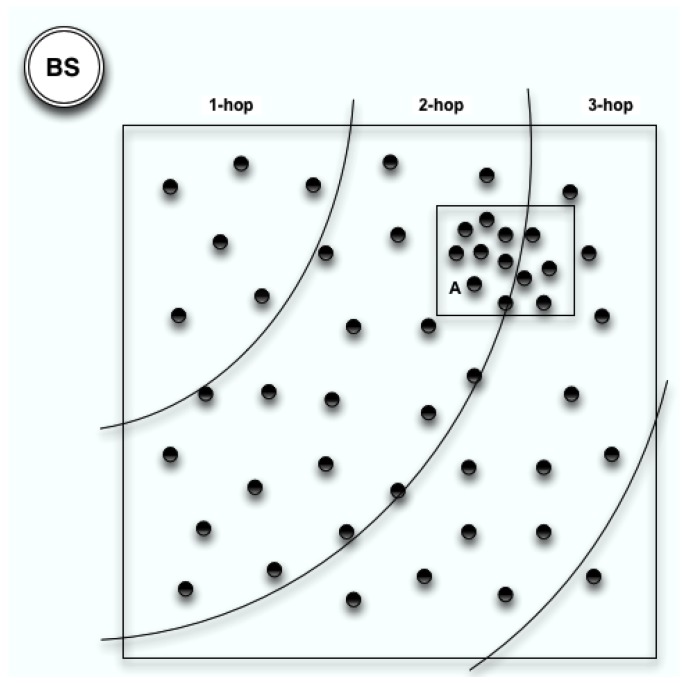
Non-uniform distributed WSN.

In a system, we believe that some of the sensors in the network are more important than the others on network coverage level. An extreme case is shown in Figure 4: area A has a higher node density than the rest of the sensing field. The nodes in area A have less impact on the coverage of the network than the nodes outside this area. The sensors that do not have a significant impact on the coverage of the network are allowed to die sooner than the others. To implement multihop communication manner, the sensors can be partitioned into layers to adopt similar strategy in CCS or S-WEB. This has motivated us to provide a distributed clustering algorithm that can not only extend network lifetime, but also guarantee the coverage of the network. The name of the algorithm is Balanced Energy-Efficient (BEE) clustering algorithm and another version supporting multihop inter-cluster communication is called Multihop BEE (BEEM).

### 3.2. Motivation for Context Awareness

As we have discussed in Section 1, clustering techniques are required and beneficial even for highly dynamic IoT systems. With 3GPP standard infrastructure, the deployment for IoT becomes more flexible and the connectivity problem can be solved straightway. When migrating IoT systems to 5G networks, several critical problems in clustering still need to be addressed.

The first challenge comes from the fundamental nature of IoT systems—the high degree of diversity. The things in the field are highly heterogeneous and some of the nodes can be extremely advanced. Comparing with traditional WSN, IoT systems are more complex and comprehensive. Traditionally the sensors in a WSN are mainly in charge of environment sensing, data collection and transmission. However, the applications in IoT systems are no longer just designed for those simple tasks. For example, the smart cameras deployed in intelligent monitoring systems are required to transmit high quality video stream to the control centre. Nowadays, in advanced IoT systems, we need to consider more complicated scenarios and user cases. The requirements for the network have raised in terms of network bandwidth and network delays. Besides, if MIMO is realised, each device will have multiple choices on communication interfaces for data transmission. Being aware of the high level application requirements and the low level hardware capabilities is essential to provide QoE supported services.

The second challenge is the cost for transmission. The energy cost is still a critical concern in IoT systems when deployed in 5G networks. In practice, LTE can be used as the default communication means. If possible (the CH is in range and the user requirements can be satisfied), some of the devices can switch to Bluetooth or ZigBee, which are much more energy efficient solutions.

Clustering techniques, in order to adapt to 5G and more complicated scenarios in IoT systems, the above challenges need to be solved. The corresponding research in those directions should be further investigated. In order to utilise MIMO 5G platform and facilitate communication in advanced IoT systems, BEEM protocol will be enhanced with a context aware feature to adapt to MIMO scenarios.

## 4. Balanced Energy-Efficient Clustering (BEE) and Multihop BEE (BEEM)

In this section, firstly we introduce the BEE protocol, which is proposed based on HEED. Then its multihop version BEEM supporting multihop inter-clustering routing is also provided.

### 4.1. Improved Iteration CH Election

In HEED, the initial probability that a sensor elects itself to be a CH is

(1) $$CH_{prob}=C_{prob}\times \frac{E_{residual}}{E_{max}}$$

$C_{prob}$ is the initial CH probability (Both LEACH and HEED set it to be 5%). $E_{max}$ is the initial energy for all the sensors. $E_{residual}$ is the remaining energy on the current sensor. If a sensor is not covered by any CHs, it will elect itself as a tentative CH when a random generated number is smaller than the value of $CH_{prob}$. After each iteration, $CH_{prob}$ is increased twice (maximal value is 1). Once $CH_{prob}$ reaches 1, a tentative sensor will declare itself as a final CH. If a sensor is not covered by any final CHs, it will declare itself as a final CH when its $CH_{prob}=1$. A sensor will terminate the iteration process when its $CH_{prob}=1$. To void really small value for $CH_{prob}$, it is set to the bigger value between $C_{prob}\times \frac{E_{residual}}{E_{max}}$ and $P_{min}=10^{-4}$. Otherwise the number of iterations can be unnecessarily large. Since $C_{prob}$ starts from 5%, a high energy sensor still needs at least 6 iterations to have a chance to declare itself as a final CH ($0.05\times 2^{N_{tier}-1}$ can be bigger than 1 only when $N_{iter}\geq 6$ where $N_{iter}$ presents the number of iterations required). As $E_{residual}$ of a sensor decreases, it needs more iterations to terminate the CH election process.

To address the first issue highlighted in Section 3, two improvements are made in the CH election process in BEE.

1. *Number of iterations:* The number of iterations to terminate the CH election process has a significant impact on the network latency and congestion. Therefore, reducing the number can reduce the overhead of the iteration CH election process.
2. *Node density:* Sensors in a high-density area are allowed to die sooner than the sensors in a low-density area for the purpose of maintaining the coverage of the network. In BEE, we believe that if a sensor’s node degree is high, it is reasonable to assume that the node density around this sensor is high. The node degree of a sensor is referred as the number of sensors in its communication range.

Based on HEED, in BEE, considering local density, $CH_{prob}$ should be calculated as:

(2) $$CH_{prob}=C_{prob}\times \frac{E_{residual}}{E_{max}}\times \min\left(\frac{Node\;Degree}{D_{avg}},1\right)$$

where $D_{avg}$ is the average density of the network and it is calculated as:

(3) $$D_{avg}=\pi R^{2}\times \frac{Num_{sensors}}{Area}$$

*R* is the default communication range of the sensors (normally it is set to the cluster radius), $Num_{sensors}$ is the total number of sensors in the network and Area refers the area of the sensing field. $D_{avg}$ is also the value for average node degree.

To reduce the number of iterations, we separate the factors in Formula (2) into three parts: $C_{prob}$, $C_{en}=\frac{E_{residual}}{E_{max}}$ and $C_{de}=\min\left(\frac{Node\;Degree}{D_{avg}},1\right)$. Three conditions that are used to determine whether a sensor can elect itself as a final or tentative CH are:

1. C1 = $\langle Random\left(0,1\right)\leq C_{prob}\rangle$
2. C2 = $\langle \frac{E_{residual}}{E_{max}}\geq 1\rangle$
3. C3 = $\langle \frac{Node\;Degree}{D_{avg}}\geq 1\rangle$

As in HEED, $C_{prob}$ is set to be the optimal CH ratio—5%. After each iteration, the value of $C_{en}$ and $C_{de}$ are both doubled. A sensor will terminate its own CH election thread when either $C_{en}$ or $C_{de}$ reaches 1. By separating the factors, we aim to decrease the number of iterations CH election process to construct the CH set. The status of the CH depends on which conditions are true as shown in Table 3. If two of the three conditions are true, the sensor will elect itself to be a final CH. If a sensor cannot communicate with any other sensors, it will elect itself to be a CH directly without executing the iteration CH election process.

### 4.2. BEE Algorithm Analysis

In BEE, each sensor has the same probability to become a tentative CH. The probability C1 remains the same after each iteration. C2 and C3 are increased after each iteration. In HEED, since the probability to become a tentative or final CH depends on $CH_{prob}$ and this value is doubled after each iteration, more sensors tend to become tentative CHs. The total number of broadcast messages in the network is the sum of the number of sensors who ever claim to be tentative CHs and the number of sensors who ever claim as final CHs. Since a tentative CH will give up being a CH when it discovers a lower cost CH in its communication range, tentative CH generation cannot help the process determine quicker. Meanwhile it increases the number of broadcast activities. Therefore, the probability to become a tentative CH should not be increased. Decomposing the probability for a sensor to become a CH into three parts can elect CHs from three different perspectives: randomly, residual energy and node density. If a node has a higher degree than the average, it always will elect itself to be a final CH and will die quickly, which supports our expectation that the sensors in a high-density area are allowed to die sooner than the sensors in a low-density area.

### 4.3. Multihop BEE—BEEM

When the network size scales, the sensors that are far away from the BS may die much earlier than the sensors close to the BS when using direct inter-cluster transmission. If the sensors do not support long distance communication, the connectivity of the network is hard to guarantee. For the above reasons, it is necessary to support multihop inter-cluster communication. Besides, multihop inter-cluster routing can further balance the power usage on each individual sensor throughout the network.

HEED declared that many multihop communication protocols could be used to guarantee a connected inter-cluster overlay topology. It provided a proof on a uniformly distributed network. However, it failed to provide the detailed implementation. Here we propose a multihop inter-cluster communication approach specialised for WSNs.

In BEEM, we adopt the same idea that is used in CCS and S-WEB in order to organise the sensors into layers as shown in Figure 4. The higher-layer CHs can transmit data to the 1-lower layer CHs. The BS will broadcast a beacon signal to all the sensors in the network at the initial stage. Based on the received single strength, the distance between a sensor and the BS can be estimated from the Log Distance Path Loss Model [33]. Based on the distance between a sensor and the BS, every sensor assigns itself a layer index. Supposedly the cluster range is Tr, and then the maximum value for the distance from a CH to its nearest CH will be 2Tr. Considering the BS as the centre point, the difference of the radius between two adjoining layers should be 2Tr. If a CH wants to transfer data back to the BS, it will transfer the data to one of the lower layer CHs first. The CH transmission range should be at least 2Tr to guarantee that it can communicate with its nearest CH. If R=2Tr, the CHs at layer *n* can transfer data to n−1 layer. If R=m×2Tr, the CHs at layer *n* can transfer data to n−m layer. In BEEM, we set R=2Tr.

## 5. Smart-BEEM For IoT Systems in 5G Scenarios

As presented in the previous section, BEEM has been proposed to extend coverage sensitive longevity for WSNs. IoT systems are going to be deployed in 5G networks when this high complex and high ability communication means can be finalised. Based on the analysis and statements in the previous sections, a clustering algorithm that can support context aware clustering in IoT systems in 5G scenarios is proposed. Based on the CH election scheme proposed in BEEM, a smart CH selection algorithm is delivered here. All together we name it Smart-BEEM. It can adapt to MIMO 5G environment and select communication interfaces and CHs according the currnt context.

### 5.1. Analysis on Existing Communication Interfaces

At present, many communication interfaces are available on IoT devices, including Zigbee, Bluetooth, WiFi, LTE, etc. New machine type communication, like LTE-M, NB-IoT and DSRC are also invented to support machine type communication. The communication interfaces all have their own characteristics, such as frequency, data rate, transmission range and energy consumption, which will also affect the throughput, network delay and in turn user utility. For example, Table 4 lists some most concerned features and the documented values for several popular communication technologies. As we can see, every single one is unique. Comparing with LTE and WiFi, Zigbee, bluetooth and NB-LTE have lower data rate. The transmission range for Zigbee, bluetooth and WiFi is shorter than NB-LTE and LTE. Apart from that, Zigbee has low energy consumption, supporting devices working for years without recharging. While the energy consumption of WiFi and LTE are relevantly high, with which IoT devices may only last for a few days.

### 5.2. Utilising MIMO

As shown in Table 4, when multiple communication interfaces are available, how to intelligently select among them for data transmission is challenging. Many existing clustering algorithms assume that all the nodes in the network are homogeneous with one single communication interface. With MIMO available in 5G networks, this assumption is no longer held. Existing work lacks flexibility and ignores the diversities of the sensor nodes. To improve existing algorithms, the context that a sensor is in should be considered. For example, a sensor should be able to choose a suitable communication interface according to its transmission requirements and its communication capability.

A new clustering algorithm that can utilise MIMO feature in 5G environment is required. Based on BEEM, Smart-BEEM is proposed. In this paper, we assume that in the system, all the IoT devices are capable of MIMO. If a node is selected as CH, it will turn on MIMO to receive data from different sources through different communication channels. Then the CHs will compress the received data and transmit the data back to the BS. Except CHs, each sensing node either transmits small volume of data or video stream (In this paper, we use the term—“*Data*” to present small volume of traffic, such as temperature or humility information; while we use the term—“*Video*” to present large volume of traffic requiring high network bandwidth, such as video stream). A sensing node will only select one communication interface and a CH for transmission through Smart-BEEM algorithm.

### 5.3. Smart-BEEM Algorithm Design

The two main objectives of Smart-BEEM are to (1) improve QoE and (2) reduce power consumption for IoT systems. Unfortunately, in most scenarios, those two objectives cannot be achieved simultaneously. In this paper, Smart-BEEM currently is designed to take QoE as the highest prioritised task. Meanwhile if possible, it will also try to reduce power consumption. For example, if a node aims to transmit temperature data back to the BS, it currently can communicate with one CH through Zigbee and another CH located further with Direct-WiFi. Since the bandwidth values for Zigbee and Direct-WiFi are both enough for temperature data, choosing Zigbee can significantly reduce energy consumption for communication. On the other hand, if the node aims to transmit video stream back to the BS and currently Direct-WiFi and Zigbee are both available, even though using Direct-WiFi will consume more energy, it will still be prioritised over Zigbee due to QoE concerns.

Each node will rank the available communication interfaces by its own preference. The ranking algorithm is described as below. If transmitting simple data, all communication interfaces are allowed. If transmitting video stream, only interfaces with NAI${}_{i}^{r}>x^{r}$ are allowed, where NAI${}_{i}^{r}$ is transmission data rate for the $i_{th}$ Network Access Interface (NAI) and $x^{r}$ is the data rate requirement from the application running on Node *x*. Then the preferred interfaces can be ranked by dividing the square of energy consumption.

(4) $$Ranking_{i}=\frac{NAI_{i}^{r}}{{\left(NAI_{i}^{e}\right)}^{2}}$$

$NAI{{}_{i}}^{e}$ is the energy consumption for $NAI{}_{i}$ over one unit transmission distance. The ranking for interface NAI${}_{i}$ is $ranking_{i}$. Supposedly Node *x* can receive CH information from *m* CHs. Then it can construct a communication table as below in Table 5:

The QoE for communication between Node *x* and connect CH${}_{j}$ through network interface NAI${}_{i}$ is defined as below:

(5) $$QoE_{i,j}=\left\{\begin{array}{cc} 0 & \;\mathrm{if}\;Node\;x\;cannot\;communication\;with\;CH_{j}\;by\;NAI_{i}\\ \frac{ranking_{i}}{\;distance_{x,j}} & \;\mathrm{otherwise}\; \end{array}\right.$$

where $distance_{x,j}$ is the distance between Node *x* and CH${}_{j}$. By checking this table, node *x* will pick the pair of NAI and CH with the highest value of QoE${}_{i,j}$.

In order to perform this algorithm, we need to do a normalisation on the features of the communication interfaces. Taking Table 4 as an example, the data rate will be classified into Low (value = 1) or High (value = 2). The energy consumption will be rated as High (value = 3), Medium (value = 2) or Low (value = 1). Any available network accessing interface in 5G communication can be added into this normalisation table.

### 5.4. Smart-BEEM Algorithm Analysis

Our former work BEEM is focusing on CH election to construct the structure for the inter-clustering communication. It elects CHs not only based on the residual energy level, but also the node density. Through this algorithm, not only the power consumption will be reduced, but also the coverage of the network can be maintained longer. However, BEEM has not specified the CH selection scheme. A Node will just choose to join the nearest CH.

Based on BEEM, Smart-BEEM proposes a CH selection by adapting to MIMO features for intra-cluster communication. Based on the communication features and requirements, each sensor will have its own preference on the network access interfaces. Firstly, a node should fulfil its responsibilities and obligations in the network. If it is supposed to transmit *Video* and then it should select an interface that has high bandwidth. On the other hand, if it is designed to transmit *Data*, it can have a larger range of selections for network interfaces. Meanwhile, the nodes also should reduce energy consumption and hence extend coverage sensitive longevity further comparing with BEEM. Hence low power consumption communication interfaces should always be prioritised when QoE is guaranteed. However, three factors should be considered before the nodes makes the final decision: (1) Application requirements on the network. (2) Transmission range of the access interface. (3) Distance between the nodes and CHs. The node itself can have its own preference on the network interfaces even only concerning its own characteristics. In this case, we refer this scenario as self-concerned approach. However, in order to select a suitable CH, the context of the CHs also should be considered. For example, considering its own interest, a node prefers to use Zigbee to transmit data. However, in its Zigbee communication range, no CHs are available. Therefore, this node has to select the secondary choice for interfaces, which has longer communication range. In another case, this node finds a CH in its Zigbee communication range. However, considering distance, the energy cost for using Zigbee is even higher than that of using NB-LTE (This might be an extreme case). At the end, NB-LTE overall may be a better choice than Zigbee. This algorithm aims to make decisions based on the requirements from the nodes themselves and the context of the network.

## 6. Experimental Construction

The proposed BEE, BEEM and Smart-BEEM algorithms will be evaluated in MATLAB simulation. In this section, the experimental settings and analysis are presented.

For different technologies in the network, we will have a normalised feature table like Table 6. In our simulation, we assume five different interfaces are available. NAI${}_{i}$ can be instanced to any technologies in Table 4 or new technologies that have not been invented yet. In the experiments, without losing generality, we use the symbolised name for the network access interfaces. There are two reasons supporting this temptation: (1) This approach makes the algorithm scalable for new network access interfaces that can be included flexibly. (2) In the future work, we will consider more complexed heterogeneous nodes, in which circumstance each node will have its own set of network access interfaces.

LEACH claims that different assumptions about the radio characteristics will affect the performance of a protocol. Two most referenced clustering algorithms, LEACH and HEED, adopted different models to compute energy consumption for long distance communication. As shown in Table 7, in LEACH, the calculation of transmission power consumption is simplified as:

(6) $$E_{Tx}=E_{elec}*k+\epsilon_{amp}*k*d^{2}$$

where *k* is message length measured in bits and *d* is the distance from the transmitter to the receiver. In the further work, LEACH research team has provided an advanced study on radio power consumption in [34]. The calculation is specified as:

(7) $$E_{Tx}=E_{elec}*k+\epsilon_{amp}*k*d^{n}$$

when $d<d_{0}$, $\epsilon_{amp}=$ 10 pJ/bit/m${}^{2}$ and *n* = 2. When $d>d_{0}$, $\epsilon_{amp}=$ 0.0013 pJ/bit/m${}^{4}$ and n=4. The value of $d_{0}$ is a constant distance that is determined by the surrounding environment. HEED has adopted this power consumption model.

Based on the power consumption model in LEACH and HEED, in this paper, three power consumption modes are defined as in Table 8. For BEEM, since only one communication interface is considered, Low and High power consumption modes are used for the default communication. For Smart-BEEM, Low, Medium and High power consumption modes are adopted, where $d_{0}$ = 40 m.

The performance of a clustering algorithm is highly related to the parameter settings in the simulation. For example, if broadcast packet has the same size as data packet, heavy broadcast will significantly undermine the performance. Besides, node density also affects the impact of broadcast. We set our simulation parameters to be the same as in HEED, shown in Table 9. 300 nodes are randomly deployed in a (0, 0) ∼ (100, 100) field with a BS located at (50, 175).

## 7. Experimental Results

In this section, firstly, we compare BEE with existing classic clustering algorithm LEACH and HEED. Then the comparison between BEE and BEEM is illustrated. At the end, we investigate the performance of Smart-BEEM.

### 7.1. Original BEE

When evaluating a clustering algorithm, power consumption is normally the only concerned metric, to the detriment of other issues such as network coverage. Coverage sensitivity longevity has been proposed to evaluate network lifetime rather than number of nodes. Network coverage is determined by the distribution and sensing range of the sensors. Some of the sensors in the network are more important than the others in terms of network coverage. The sensors that do not have a significant impact on the coverage of the network are allowed to die sooner than the others. The experimental results for original BEE are shown below.

#### 7.1.1. Network Distribution

**Figure 5:**
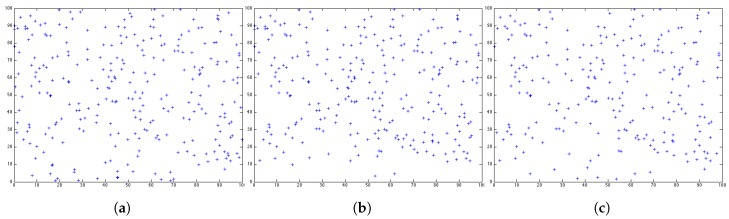
Sensor distribution after 450 rounds using LEACH, HEED and BEE. (**a**) LEACH; (**b**) HEED; (**c**) BEE.

Figure 5c shows after 450 rounds performing BEE, the snapshot for the distribution of the sensors still alive in the network. Comparing with the distributions for LEACH and HEED, BEE can provide better coverage at 450 round, with the same number of nodes as LEACH and less number of nodes comparing with HEED. The exact value for coverage is will be discussed in detail later.

#### 7.1.2. CH Rate

**Figure 6:**
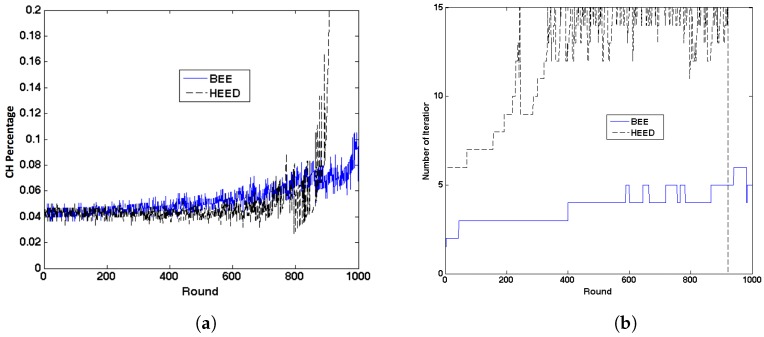

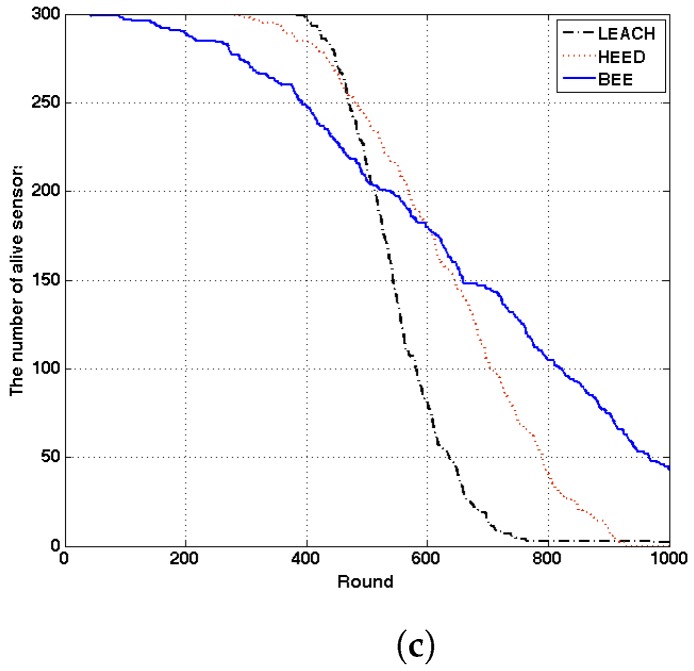
Comparison between HEED and BEE. (**a**) CH percentage; (**b**) The iterations required in each round; (**c**) Number of sensors still alive.

Comparing the CH percentage in Figure 6, we can see that BEE has similar number of CHs as HEED. In HEED, the CH percentage increased to 1 at the end. This is because all the sensors still alive were no longer in each other’s cluster range and then every sensor was required to be CH to transmit data directly to the BS. It has been proved that in HEED the elected CHs can be well distributed in the network and cover the whole sensing area. The case that two CHs in each other’s cluster range is rare. Since BEE does not need more CHs than HEED, the CHs elected through improved iteration CH election process in BEE can also cover the whole sensing area.

#### 7.1.3. Clustering Iterations

The number of iterations in the clustering forming phase determines the overhead of the algorithm. Reducing the number of required iterations in each round can reduce (1) the clustering delay and (2) the number of broadcast packets. The comparison of the number of iterations in each round is shown in Figure 6b. By separating $CH_{prob}$ into three parts, the number of iterations in each round was significantly reduced in BEE. At the end, for HEED, the number of iterations suddenly dropped to 0 because no sensor was still alive in the network. The number of iterations of HEED can increase from 6 to 15. When the battery level is high, as we mentioned in Section 4.1, 6 iterations are necessary since $C_{prob}=5\%$. When the battery level is low, it takes 15 iterations as $P_{min}=10^{-4}$. However, the number of iterations for BEE will not increase significantly as the sensor batter level decreases.

#### 7.1.4. Longevity

The number of sensors still alive sensors in the network is shown in Figure 6c. In the period from 0 to 450 round, more sensors died in BEE than in HEED and LEACH. After 600 round, BEE had more sensors left in the network. In long term, BEE has longer network lifetime than HEED and LEACH. In the next section, we will prove that the sensors died at the beginning have little impact on the coverage of the network.

#### 7.1.5. Network Coverage

**Figure 7:**
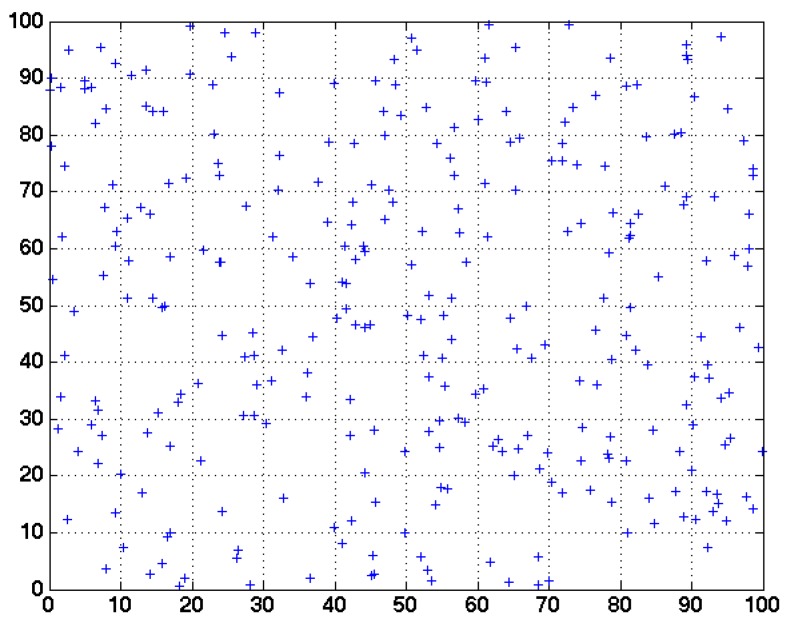
Sensing grid of the network at the initial state.

**Figure 8:**
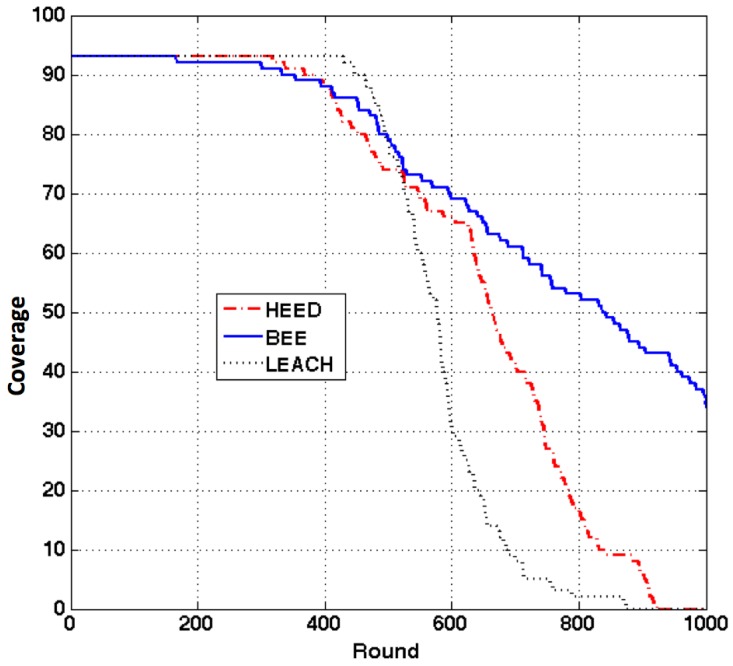
Network sensing coverage.

The cluster radius in both BEE and HEED is set to be 25 m. The sensing radius is set to be 10 m. To simplify our problem, we partition the network area into grids of the size 10 m × 10 m rather than circles. Through this way, the sensing field is partitioned into 100 cells. In each cell, if there is a sensor still alive, we assume this area can be monitored by system. To guarantee the network coverage, a clustering algorithm should be able to cover the cells maximally. Since the sensors are randomly deployed in the network, the initial coverage with 300 sensors is 93 cells, as shown in Figure 7. The coverage of the network after each round is shown in Figure 8.

We can see from Figure 6c that nodes started to die around 100 round for BEE. However, in Figure 8, BEE started to lose coverage around 180 round. It means that the sensors started to die from 100 round till 180 round did not have any impact on the sensing coverage of the network. For long term, BEE can provide better coverage than HEED and LEACH.

#### 7.1.6. Multihop Inter-Clustering Supported BEE (BEEM)

**Figure 9:**
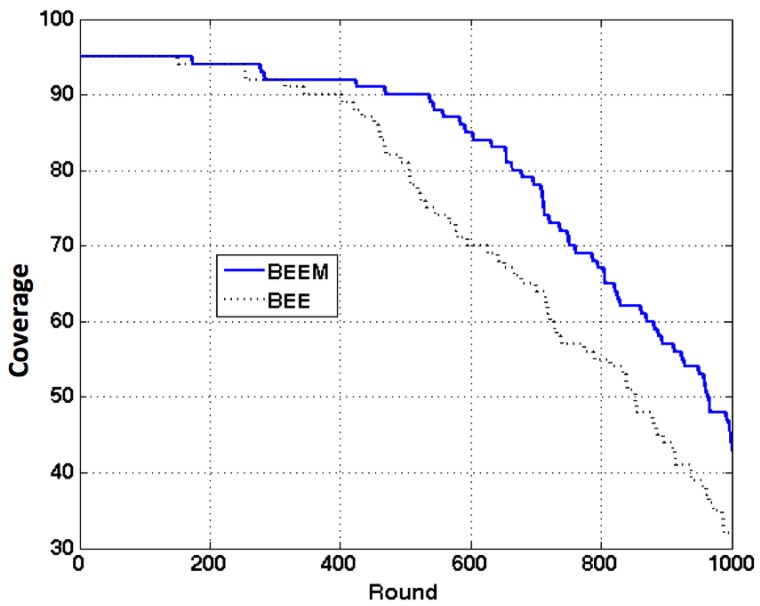
Network sensing coverage comparison between BEE and BEEM.

The communication between the CHs and the BS is called inter-cluster communication. When the network size scales, if the sensors do not support long distance communication, the connectivity of the network is hard to guarantee without multihop routing. Because of that, supporting multihop inter-cluster communication is essential. Furthermore, multihop inter-cluster routing can further reduce energy consumption through reducing long distance communication between the CHs and the BS. The experimental results for BEEM are shown in Figure 9. The energy cost of forwarding packets is set to be 10 pJ/bit/m${}^{2}$. As we can see, BEEM can provide better network coverage and longer overall lifetime than BEE.

### 7.2. Comparing between BEEM, Self-Concern Solution and Smart-BEEM

Since it is normally assumed that only one communication interface exists in the traditional WSNs, choosing the nearest CH can maximally reduce the power consumption on the sensor nodes. Same as most existing work, one assumption in BEEM for the network is that

All the nodes are homogeneous and have similar communication and computation capabilities.

This assumption can be seen in most studies for clustering. However, it will no longer be true in the high dynamic IoT systems. When the sensors are able to communicate through MIMO channels, we must be able to modify BEEM to adopt to this feature. In this section, we will illustrate the benefit for utilising this feature. Three approaches are analysed and compared:

1. **Original BEEM:** Ignoring the MIMO feature, the sensors always use the communication interface with the highest bandwidth to guarantee the QoE for both *Video* and *Data* transmission. The nodes still choose the nearest CH to join. If no CHs are in the default cluster radius range, the nodes will transmit date back to the BS directly.
2. **Self-concerned BEEM:** In this approach, the nodes are aware of the multiple communication interfaces. However, each node will only choose its most preferred communication interface. If no CH in the range of this communication interface is available, it will halt the CH selection process and transmit data back to the BS directly using high power long distance communication (NAI${}_{5}$).
3. **Smart-BEEM**: BEEM algorithm is carried out firstly to elect CHs. Based on Smart-BEEM algorithm, each node selects a communication interface and CH with the highest QoE value. If no CHs are in range, same as above, the node will transmit data back to the BS directly though the default interface—NAI${}_{5}$.

**Figure 10:**
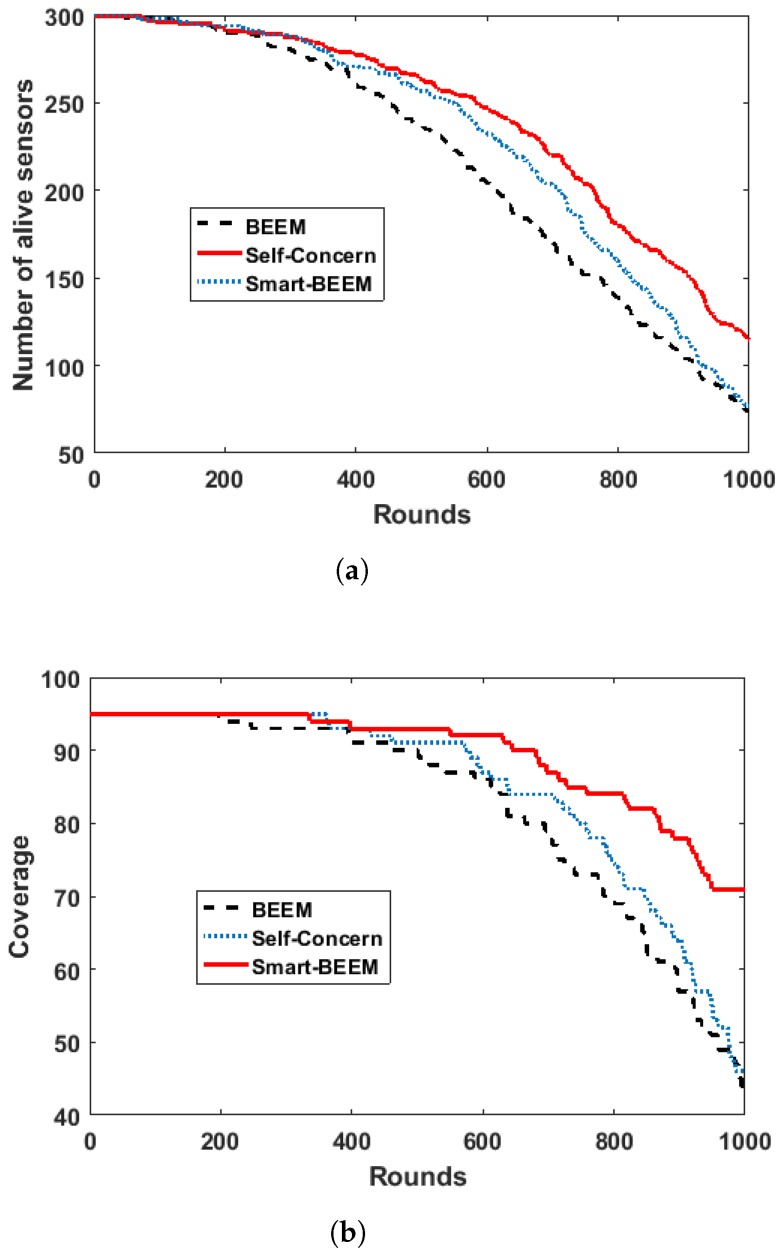
Comparison between BEEM, Self-Concerned and Smart-BEEM. (**a**) Number of sensors still alive; (**b**) network coverage.

As described before, Self-Concerned BEEM and Smart-BEEM are both based on BEEM. BEEM is focusing on CH election. Self-concerned BEEM and Smart-BEEM aim to facilitate IoT nodes to select communication interfaces and CHs according the current context. As we can see from Figure 10a, Self-Concerned BEEM can maintain the number of alive sensors better than the original BEEM. Furthermore, Smart-BEEM exceeds both original BEEM and Self-Concerned BEEM in terms of maintaining the number of alive sensors. Figure 10b has shown that Self-Concerned BEEM has maintained the coverage slightly better than the original one. Smart-BEEM has significantly increased the coverage at each round comparing with the other two solutions. When applying the original BEEM clustering algorithm in the network, the MIMO feature was ignored by the applications. In order to guarantee QoE, communication interfaces with high bandwidth were always prioritised. Since some of the sensors were only transmitting simple data information, energy consumption can be reduced through using low energy consumption interface if the sensors could be aware of the MIMO feature. Self-concerned BEEM can be aware of the requirements from the application and also the MIMO features. It can select suitable interface based on its own requirements. Therefore, its performance was better than the original BEEM. Further improved based on Self-BEEM, Smart-BEEM can also be aware of the context of the network. It can outperform Self-BEEM by selecting more suitable CHs for more energy efficient routing. The experimental results have proved our point of view—the ability of context awareness can further improve QoS and QoE When a sensor is aware of (1) the application requirements, (2) the MIMO feature and (3) network context, it can make the best decision for data transmission.

## 8. Future Work

There are several perspectives that our design can be further advanced to cater for heterogeneous IoT systems. Firstly, in this paper, the IoT devices in the network are assumed with the same level of energy storage. This is normally not true in most real systems. The CH election process in BEEM has counted node energy level as one factor. When the nodes selecting CHs, their own energy levels should also be considered. Some of the nodes in the IoT systems are even rechargeable or pre-deployed with unlimited power supply, which should affect the design of the algorithm.

Secondly, in our current study, all the IoT devices have equipped with the same 5 communication interfaces. In the future work, each sensor is assumed to have a random ability for network access interfaces. The combination can be {NAI${}_{1}$, NAI${}_{5}$}, {NAI${}_{2}$, NAI${}_{4}$, NAI${}_{5}$} or {NAI${}_{5}$}. NAI${}_{5}$ is a default one for every node. Furthermore, other communication interfaces with characteristics that are different from the above 5 ones can also be considered. With the fast development for radio technologies, new interfaces are highly expected in the future. For example, an interface that allows low rate and high communication range can also be considered in our solution.

Thirdly, the price policy for different communication technologies can be another concern. Data transmission on licensed bands normally more expensive than that on unlicensed bands. User requirements on financial cost can also have influence on the interface selection.

Fourthly, in this paper, user utility has the highest priority when selecting CHs and communication interfaces. However, in real systems, power consumption and user utility should be balanced well. In some specific circumstance, user utility may be sacrificed to gain more energy efficiency.

## 9. Conclusions

Clustering techniques are proposed to provide a platform for network topology management to extend network lifetime. Since most existing clustering algorithms overlook the importance of network coverage when evaluating the performance, firstly we have introduced a new metric to measure network lifetime—coverage sensitive longevity. Then we have proposed a Balanced Energy-Efficiency clustering algorithm (BEE) to extend coverage sensitive longevity. Its multihop version supporting mutihop inter-cluster communication, called multihop BEE (BEEM) is also provided. It can maintain coverage notably comparing with classic clustering algorithms—LEACH and HEED.

Secondly, we have also presented the development trend from traditional WSNs to advanced IoT systems, especially in future network scenarios. In order to cater for dynamic IoT systems deployed in 5G communication environment, we have further enhanced BEEM algorithm with smart intra-cluster routing scheme and named it as Smart-BEEM. Smart-BEEM can adapt to the MIMO communication feature to further improve QoS and QoE. It can facilitate advanced IoT devices to select communication interfaces and CHs based on the network context, including characteristics of the network access interfaces, the current user usage and the details of the available CHs. The experimental results have shown that Smart-BEEM can better maintain the number of alive nodes and the network coverage comparing with BEEM.

At the end, we have delivered a discussion on the future work that can further improve our algorithm in order to be deployed in more realistic systems from the following perspectives: (1) Diverse energy storage situation. (2) Heterogeneous communication interfaces on nodes. (3) Financial cost and (4) Balancing between energy efficiency and QoE.

## Figures and Tables

**Table 1:** Comparison of 6 Existing Clustering Algorithms and BEE(M).

Algorithm	Cluster Structure	Distributed	Node Density	CH Rotation	Data Fusion	TPC	Energy Aware	Cluster Size	Sensing Abilities	Simulation	Compared with
LEACH	Voronoi	Yes	No	Yes	Yes	Yes	No	No	None	MATLAB	Direct MTE, Static
HEED	Voronoi	Yes	Yes	Yes	Yes	Yes	Yes	Yes	Energy	MATLAB	LEACH
DWEHC	Voronoi	Yes	Yes	Yes	Yes	Yes	Yes	Yes	Location Energy	NS-2	HEED
PEGASIS	Chain	Yes	No	Yes	Yes	No	Yes	No	Location	MATLAB	Direct LEACH
CCS	Chain	No	No	Yes	Yes	No	Yes	No	Location Energy	MATLAB	PEGASIS
S-WEB	Spectrum	No	No	No	No	No	Yes	No	Location Energy	MATLAB	Direct
BEE(M)	Voronoi	Yes	Yes	Yes	Yes	Yes	Yes	Yes	Energy	MATLAB	LEACH HEED

**Table 2:** Summaries for Selected Existing Survey Papers on Clustering for WSNs.

Survey	Major Contributions
Ameer [24] 2007	1. Earlier clustering work before LEACH mainly addresses node failure problem.
	2. Clustering algorithms after LEACH start to focus more on energy efficiency.
	3. Multihop intra-cluster topology is rare.
	4. Multihop inter-cluster communication is well applied.
Afsar [26]	1. Energy efficiency/maxing lifetime is the dominate objective for clustering.
	2. No reviewed clustering algorithm can operate in a heterogeneous network.
	3. Multihop intra-cluster communication is rarely supported.
	4. Multihop inter-cluster communication is well applied.
	5. The CH is only in charge of data aggregation and transmission.
	6. Device mobility is hardly concerned in existing solutions.
	7. Distributed implementation is the mainstream in clustering.
Liu [25,27]	1. Algorithms have small deliver delays tend to have low energy efficiency.
2012	2. Distributed implementation is the mainstream in clustering.
2015	3. Tree and train based implementations have lower scalability than grid based ones.

**Table 3:** CH Self-election Table.

C1	C2	C3	CH Status
1	1	1	Final
0	1	1	Final
1	0	1	Final
1	1	0	Final
1	0	0	Tentative

**Table 4:** Documented Characteristics for Different Communication Interfaces.

Communication Means	Bandwidth	Data Rate	Transmission Range	Energy Consumption
WiFi (802.11a)	2.4 GHz; 5 GHz	54 Mbps	30 m	Medium 15–20 dBm
Bluetooth	2.4–2.5 GHz	1 Mbps	10 m	Low Days
Zigbee	868–915 MHz; 2.4 GHz	250 kbps	10–100 m	Low Years
LTE	2.6 GHz	Uplink: 5 Mbps Downlink: 10 Mbps	<15 km	23 dBm
NB-LTE	180 kHz	Uplink: 250 kbps Downlink: 170 kbps	<15 km	20–23 dBm

**Table 5:** Communication Table for Node *x*.

Communication Interface	CH${}_{1}$	CH${}_{2}$	...	CH${}_{m}$
NAI${}_{1}$	QoE${}_{1,1}$	QoE${}_{1,2}$	...	QoE${}_{1,m}$
NAI${}_{2}$	QoE${}_{2,1}$	QoE${}_{2,2}$	...	QoE${}_{2,m}$
...	...	...	...	...
NAI${}_{n}$	QoE${}_{n,1}$	QoE${}_{n,2}$	...	QoE${}_{n,m}$

**Table 6:** Normalised Feature Values for Different Communication Interfaces.

Communication Means	Data Rate	Transmission Range	Energy Consumption
NAI${}_{1}$	Low	10 m	Low
NAI${}_{2}$	Low	20 m	Low
NAI${}_{3}$	Low	40 m	Medium
NAI${}_{4}$	High	25 m	High
NAI${}_{5}$	High	1 km	High

**Table 7:** Energy Dissipated for LEACH and HEED.

Protocols	Transmission/Receiving ($E_{\mathrm{elect}}$)	Transmit Ampilier ($\epsilon_{\mathrm{amp}}$)
LEACH	50 nJ/bit	100 pJ/bit/m${}^{2}$
HEED	50 nJ/bit	10 pJ/bit/m${}^{2}$ ($d<d_{0}$)
		0.0013 pJ/bit/m${}^{4}$ ($d>d_{0}$)

**Table 8:** Energy Dissipated For BEEM and Smart-BEEM.

Power Consumption Mode	Transmission/Receiving ($E_{\mathrm{elect}}$)	Transmit Amplifier ($\epsilon_{\mathrm{amp}}$)
Low Power Consumption	50 nJ/bit	10 pJ/bit/m${}^{2}$ ($d<d_{0}$)
Medium Power Consumption	50 nJ/bit	15 pJ/bit/m${}^{2}$ ($d<d_{0}$)
High Power Consumption	50 nJ/bit	0.0013 pJ/bit/m${}^{4}$ ($d>d_{0}$)

**Table 9:** Simulation Parameters.

Type	Parameters	Values
Network	Network area	From (0, 0) to (100, 100)
	Sink	At (50, 175)
	Number of Sensors	300
	Initial Energy	0.5 J
Application	Default cluster radius	25 m
	Data packet size	100 bytes
	Broadcast packet size	25 bytes
	Data header size	25 bytes
	Each round	5 TDMA frames
	CH data compress rate	0.8
Simulation	Duration	1000 rounds
	Default acess interface	NAI${}_{5}$

## Abbreviations

The following abbreviations are used in this manuscript:

CH	Cluster Header
BS	Base Station
MIMO	Multiple-In Multiple-Out
